# Psychotropic medication use among elderly nursing home residents in Slovenia: cross-sectional study

**DOI:** 10.3325/cmj.2011.52.16

**Published:** 2011-02

**Authors:** Marija Petek Šter, Eva Cedilnik Gorup

**Affiliations:** Department of Family Medicine, Medical faculty, University of Ljubljana, Ljubljana, Slovenia

## Abstract

**Aim:**

To determine the prevalence of psychotropic medication prescribing in elderly nursing home residents in Slovenia and to explore the residents’, physicians’, and nursing home characteristics associated with prescribing.

**Methods:**

In a cross-sectional study, we collected the data for 2040 nursing home residents aged 65 years and older in 12 nursing homes in Slovenia between September 25 and November 30, 2006. Prescribed medications lists were retrieved from patients’ medical records. Psychotropic medications were coded according to Anatomical Therapeutic Chemical Classification 2005, which we adjusted for the purposes of the study. Multivariate logistic regression analysis was performed to determine the residents’, physicians’, and nursing home characteristics associated with prescribing.

**Results:**

Residents were from 65 to 104 years old (median, 83 years) and 1606 (79%) of them were female. A total of 970 (48%) residents had dementia and 466 had depression (23%). In 1492 (73%) residents, at least one psychotropic medication was prescribed. Nine hundred sixty residents were prescribed hypnotics and sedatives (47%), 572 (28%) antipsychotics, 460 (23%) antidepressants, and 432 (21%) anxiolytics. Residents’ characteristics associated with psychotropic medication use were female sex (odds ratio [OR], 1.36; 95% confidence interval [CI], 1.03-1.80), age (OR, 0.97; 95% CI, 0.95-0.98), permanent restlessness (OR, 2.54; 95% CI, 1.71-3.78), dementia (OR, 1.76; 95% CI, 1.33-2.34), depression (OR, 5.51; 95% CI, 3.50-7.58), and the number of prescribed medications (OR, 1.29; 95% CI, 1.23-1.35). Of physicians’ characteristics (sex, age, specialization in general practice, years of working experiences as a general practitioner, and years of experiences working in a nursing home), male sex was associated with psychotropic medication prescribing (OR, 1.80; 95% CI, 1.17-2.76).

**Conclusion:**

Frequency of psychotropic medication prescribing in elderly nursing home residents in Slovenia is high and is comparable to Western European countries. Our next step should be optimizing the prescribing in patients with the highest prescription rate.

Nursing homes in Slovenia are non-profit state-owned or private institutions for social care. They mainly offer help in everyday activities, nursing, treatment, and rehabilitation to the elderly who are incapable of living at home due to old age, illness, or other reasons. The residents are also attended by a general practitioner, who is a private contractor or employed in state-owned primary care health centers.

The number of nursing homes and nursing home residents in Slovenia has increased in the recent years and now there are 94 nursing homes with 19 000 residents, which means there are 44 places in nursing homes per 1000 population aged 65 or over (1). The number of places in nursing homes in Slovenia is comparable to the average number of long-term beds in European countries, which varies from 14 in Italy, 48 in Germany, to 70 in Sweden (2).

Several studies have shown that nursing home residents represent a frail population, exposed to polypharmacy (taking 6 or more drugs at once) (3) and potentially inappropriate medication use (4-9). The most frequently used criteria for potentially inappropriate medication use in older adults are the Beers criteria (10), which list medications, classes of medications, and combinations of specific disease/conditions and medications to be avoided.

The group most exposed to inappropriate prescribing are residents taking psychotropic medications, as well as residents regularly taking 9 or more drugs. Psychoactive drugs accounted for 38% of all medication problems, and multiple psychoactive drugs were considered particularly problematic (11). The class which is most often involved in medication problems are antipsychotics, which are often used for intractable behavioral symptoms of dementia or psychosis unresponsive to non-pharmacologic interventions. Treatment with antipsychotics has been associated with serious adverse events (12). In addition to well described adverse effects such as sedation, falls, and extrapyramidal and anticholinergic symptoms, atypical and typical antipsychotics may also increase the risk of death in older adults with dementia (13-15). Benzodiazepines (with no differences between short and long-acting) and antidepressants (mainly tricycle antidepressant) increase the risk of falls and fractures (16-19).

The prevalence of psychotropic medications in nursing home residents varied substantially between studies, from 50 to 80%, depending on the setting and country (20-23). Determinants of psychotropic medication prescribing in nursing homes are also various and depend on the country, and include permanent restlessness (21), diagnosis of dementia (17), prior treatment with these drugs at home and talking to nurse/physician about problems with anxiety, insomnia or depression (24), and nursing home characteristics (25).

The use of psychotropic medication should be critically evaluated in nursing homes in Slovenia but no data are currently available on this issue. The aim of our study is to determine the prevalence of psychotropic medication prescribing in elderly nursing home residents in Slovenia and to explore the residents’, physicians’, and nursing home characteristics associated with prescribing. We also aim to determine the frequency of incorrect or suboptimal prescribing.

## Participants and methods

### Study design

In this cross-sectional study, we invited 15 general practitioners working in nursing homes who were members of the Elderly People Workgroup (a part of Slovenian Family Medicine Society) and 13 (87%) of them agreed to participate. The participating physicians were from 12 nursing homes: Trebnje, Mengeš, Ljubljana-Kolezija, Ljubljana-Bokalce, Šentjur, Kamnik, Cerknica, Medvode, Ljubljana-Tabor, Preddvor, Krško, and Izola.

Nursing homes are located in all regions of Slovenia, most of them in small towns. The average nursing home in Slovenia has between 150 and 200 residents, but some of the nursing homes in the two main cities have more. The biggest nursing homes in Ljubljana (Bokalce, Tabor-Poljane) have up to 500 residents and the biggest nursing home in Maribor (Dom Danice Vogrinec) has up to 800 residents, accommodated in 2 to 3 buildings (1).

The study took place between September 25 and November 30, 2006 and received the National Medical Ethics Committee approval.

### Participants

General practitioners collected the data for all the residents in the nursing homes with up to 200 residents or for every other resident according the alphabetical order in the nursing homes with more than 200 residents. The residents signed the informed consent, and if they were unable, their relatives were asked to do it.

Of 2222 nursing home residents, we excluded 151 (6.8%) who were younger than 65 years, 3 refused to participate, and for 18 we were unable to obtain the informed consent. We excluded further 10 residents because of a lack of data about drug use, which left the final sample of 2040 residents.

Comparison of basic characteristics (age, functional status) between the nursing home residents from our study and nursing home residents in Slovenia (26) showed that our population was representative of the entire country.

### Data collection

The data about participating nursing home residents were obtained using a questionnaire. The questionnaire consisted of questions about demographic data (sex, age), functional status, diagnoses important for medication prescribing according to Beers criteria considering diagnosis (10), and data about drug prescribing.

Data were obtained from paper (data about diagnosis and functional status) and electronic medical records (data about drug prescribing).

According to the functional status, we divided the residents into 3 categories based on the definition of the National Health Insurance Company (27):

1. Independent in daily activities: do not need any help in basic daily activities.

2. Partly dependent in daily activities: need some help in basic daily activities, for example help in maintaining hygiene, dressing, putting on shoes, or walking.

3. Bedridden: permanently lie in bed and are completely dependent on caretakers in basic daily activities.

The term permanent restlessness means the need for 24-hour constant monitoring due to the intractable behavioral symptoms of the resident (27).

Psychotropic medications were coded according to the Anatomical Therapeutic Chemical Classification 2005 adjusted for the purpose of the study as listed: N 6 – antipsychotics (promazine, fluphenazin, haloperidol, clozapine, quetiapine, sulpiride, risperidone, olanzapine); N 7 – anxiolytics (alprazolam, bromazepam, diazepam, clobazam, lorazepam, medazepam, oxazepam); N 8 – antidepressants (tricyclic antidepressants, selective serotonin reuptake inhibitors [SSRI] class drugs, monoamine oxidase inhibitors type A, others: bupropion, duloxetine, venlafaxine, tianeptine, mianserin); N 9 – hypnotics and sedatives (zolpidem, midazolam, flurazepam, nitrazepam); N10 – antidementives (donepezil, galantamine, rivastigmine, memantine).

The term “psychotropic medication” includes antipsychotic drugs, anxiolytics, antidepressants, hypnotic drugs, or sedatives.

In a separate form, we collected the data about the general practitioners and their experience in working in nursing homes and the characteristics of nursing homes. Detailed data about the prescribed medications and potentially inappropriate prescribing in nursing home residents in Slovenia were published previously (28).

We examined the residents’, physicians’, and nursing home characteristics associated with the prescription of any of the psychotropic medication and prescription of antipsychotics and benzodiazepines. We did a separate analysis for the two groups of psychotropic medications which are, according to the data from literature and expert opinions (13-18), the main source of prescribing mistakes: antipsychotics, which are highly prescribed in people with dementia and due to the serious side effects should not be used for treatment of behavioral symptoms of dementia (29), and benzodiazepines, which are also frequently prescribed and might result in falls. Falls are the dominant cause of injury among elderly people and the reason for half of deaths due to the injuries in the elderly people (30).

### Statistical analysis

For statistical analysis, we used SPSS, version 17.0 (SPSS Inc., Chicago, IL, USA). The statistical significance level was set at *P* < 0.05. Descriptive statistics was performed to describe baseline residents’, general practitioners’, and nursing home characteristics and to describe psychotropic drug prescribing in nursing home residents. Data were presented using median and range, and proportions using percentages. To identify differences between different variables, we used independent samples *t*-test, χ^2^ test, and one-way ANOVA.

Multivariate logistic regression analysis was performed to investigate residents’, physicians’, and nursing home characteristics associated with psychotropic medication prescription. The parameters describing psychotropic medication prescription (at least one psychotropic medication, antipsychotics, and anxiolytics) were considered as outcomes. Logistic regression models were fitted separately with regard to 3 different dependent variables: at least one psychotropic medication, antipsychotics, and anxiolytics.

## Results

### Study population – residents, physicians, and nursing homes

*Residents.* We analyzed the data for 2040 residents, 1606 (79%) of whom were female. Data about functional status (level of help needed and mobility) were obtained for only 1707 residents. There were 1492 residents (73%) who were taking psychotropic medications and 548 (27%) residents who were not taking psychotropic medication. Table 1 shows baseline characteristics of the residents, stratified by psychotropic medication. There were 1032 (51%) residents who were taking 6 or more drugs at the same time, indicating polypharmacy, and 1242 (61%) residents with the diagnosis of dementia or depression or both.

**Table 1:** Baseline characteristics of the residents in nursing homes in Slovenia, stratified by psychotropic medication use

		Psychotropic medication†		
Characteristics	All residents (n = 2040)	yes (n = 1492)	no (n = 548)	P* (*t*-test or χ^2^ test)
**Age**, median (range)	83 (65-104)	82 (65-104)	83 (65-102)	<0.001
**Sex, n (%):**				0.043
male	434 (21.3)	301 (69.4)	133 (30.6)	
female	1606 (78.7)	1191(74.2)	415 (25.8)	
Diagnosis				
permanent restlessness	553 (27.1)	465 (84.1)	88 (15.9)	<0.001
dementia	970 (47.5)	775 (79.9)	195 (20.1)	<0.001
depression	466 (22.8)	460 (98.7)	6 (1.3)	<0.001
Number of prescribed drugs (median/range)	6 (0-22)	6 (1-22)	4 (0-14)	<0.001
Functional status:^‡^	all residents (n = 1707)	yes (n = 1217)	no (n = 490)	
needs no help in daily activities	474 (27.8)	303 (63.9)	171(36.1)	<0.001
needs help in most daily activities	500 (29.3)	381(76.2)	119 (23.8)	0.068
bed-ridden	733 (42.9)	533 (72.7)	200 (27.3)	0.747

*t-test or χ^2^ test.

†The percentages in brackets are calculated from the total sample.

‡Due to the lack of data on functional status assessment in one of the three categories according to the definition of the National Health Insurance Company (27), the data were calculated for a smaller number of residents.

*Nursing homes.* The number of residents in the participating nursing homes ranged from 147 to 394, median 223. Nursing homes were located from less than 1 km to 11 km (median, 1 km) from the nearest health center and less than 1 km to 50 km (median 13 km) from the nearest hospital.

*Physicians.* Physicians’ age was from 33 to 52 years, median 37 years; 10 physicians (77%) were female. Eight physicians (61%) were specialists in general practice and 5 (39%) were residents in general practice. They were working as general practitioners from 3 to 26 years (median, 10 years) and were working as general practitioners in nursing homes from 2 to 16 years (median, 4 years).

### Prevalence of psychotropic drug medication use

Psychotropic medication use is shown in Table 2. Out of 970 patients with dementia, 266 (27%) were prescribed antidementives and 423 (44%) antipsychotics. Hundred and seventeen (44%) patients with dementia who were prescribed antidementives were also prescribed antipsychotics. Two hundred and twelve (22%) patients with dementia were prescribed antidepressants in addition to other therapy.

**Table 2:** Psychotropic medication use among the residents of nursing homes (n = 12) in Slovenia in 2006

Psychotropic medication	No. (%) of residents
Any psychotropic medication	1492 (73.1)
Antipsychotics	572 (28.0)
Anxiolytics	432 (21.1)
Hypnotics and sedatives	960 (47.1)
Antidepressants	460 (22.5)

Out of 466 patients with depression, 460 (99%) were prescribed antidepressants. Two hundred and six (21%) patients with dementia and 125 patients with depression (27%) were prescribed anxiolytics. In 140 patients taking antipsychotics and in 117 patients taking anxiolytics, indication for prescribing could not be derived.

### Association of psychotropic medication prescription with residents’, physicians’, and nursing home characteristics

Results of logistic regression models are presented in Table 3; all variables included in the models are presented.

**Table 3:** Logistic regression for the use of any psychotropic medication (PM), anxiolytics and antipsychotics in the residents of nursing homes in Slovenia*

	Any psychotropic medication^†^			Anxiolytics^‡^			Antipsychotics^§^		
	B±SE	*P*	OR (95% CI)	B±SE	*P*	OR (95% CI)	B±SE	*P*	OR (95% CI)
Constant	1.720	0.118		-4.003	0.001		1.068	0.303	
Sex-female	0.309 ± 0.143	0.031	1.36 (1.03-1.80)	0.385 ± 0.155	0.013	1.47 (1.09-1.99)	-0.68 ± 0.138	0.623	0.93 (0.71-1.22)
Age of the resident	-0.034 ± 0.008	<0.001	0.97 (0.95-0.98)	-0.025 ± 0.008	0.003	0.97 (0.96-0.99)	-0.029 ± 0.008	<0.001	0.97 (0.96-0.99)
Needs no help in daily activities	-0.252 ± 0.255	0.325	0.80 (0.48-1.33)	-0.178 ± 0.251	0.478	1.35 (0.82-2.23)	0.236 ± 0.232	0.308	1.27 (0.80-2.00)
Needs help in most daily activities	0.072 ± 0.240	0.765	0.78 (0.47-1.28)	0.269 ± 0.229	0.239	1.49 (0.95-2.33)	0.132 ± 210	0.513	1.41 (0.77-1.69)
Bed- ridden	0.013 ± 0.230	0.954	1.07 (0.67-1.72)	0.124 ± 0.221	0.574	1.29 (0.84-1.98)	0.350 ± 0.188	0.062	1.42 (0.98-2.05)
Permanent restlessness (needs constant monitoring)	0.933 ± 0.202	<0.001	2.54 (1.71-3.78)	0.601 ± 0.191	0.002	1.84 (1.26-2.65)	1.495 ± 0.169	<0.001	4.46 (3.20-6.21)
Dementia	0.556 ± 0.145	<0.001	1.76 (1.33-2.34)	0.051 ± 0.144	0.722	1.05 (0.79-1.40)	0.670 ± 0.134	<0.001	1.95 (1.50-2.54)
Depression	1.639 ± 0.197	<0.001	5.15 (3.50-7.58)	0.305 ± 0.141	<0.001	1.38 (1.03-1.79)	0.008 ± 0.139	0.956	1.01 (0.77-1.32)
Number of all drugs regularly taken	0.254 ± 0.023	<0.001	1.29 (1.23-1.35)	0.180 ± 0.020	<0.001	1.18 (1.15-1.25)	0.080 ± 0.019	<0.001	1.08 (1.04-1.13)
Number of residents in the nursing home	0.002 ± 0.001	0.047	1.00 (1.00-1.00)	0.005 ± 0.001	<0.001	1.01 (1.00-1.01)	0.000 ± 0.001	0.601	1.00 (1.00-1.00)
Distance to the nearest hospital	-0.010 ± 0.022	0.659	1.00 (0.95-2.76)	0.031 ± 0.23	0.173	1.01 (0.99-1.03)	-0.013 ± 0.009	0.135	0.99 (0.97-1.00)
Sex of the physician (male)	0.586 ± 0.219	0.007	1.80 (1.17-2.76)	0.959 ± 0.232	<0.001	2.61 (1.65-4.11)	-0.136 ± 0.215	0.526	0.87 (0.57-1.33)
Age of the physician	-0.022 ± 0.011	0.061	0.98 (0.96-1.01)	0.046 ± 0.012	<0.001	1.05 (1.02-1.07)	-0.021 ± 0.010	0.039	0.98 (0.96-1.00)
Physician’s working experience in nursing home	-0.047 ± 0.027	0.830	0.94 (0.91-1.05)	-0.116 ± 0.026	<0.001	0.89 (0.85-0.94)	-0.052 ± 0.025	0.036	0.95 (0.91-1.00)
Specialization in general practice	-0.198 ± 0161	0.241	0.83 (0.60-1.14)	0.206 ± 0.198	0.299	1.23 (0.83-1.81)	0.083 ± 0.176	0.639	1.09 (0.77-1.53)

*Abbreviations: SE – standard deviation; OR – odds ratio; CI – confidence interval.

†Logistic regression. Model χ^2^_15_ = 397.492, *P* < 0.001, R^2^ = 0.271.

^‡^Logistic regression. Model χ^2^_15_ = 188.850, *P* < 0.001, R^2^ = 0.145.

^§^Logistic regression. Model χ^2^_15_ = 277.676, *P* < 0.001, R^2^ = 0.193.

Psychoactive medications were more often prescribed to female, younger residents, permanently restless, residents with dementia and depression, residents with more regularly prescribed drugs, in bigger nursing homes, and by male physicians. In fact, the odds that a female patient will be prescribed psychoactive medication were 1.46 times higher than the odds in her male counterpart. Also, patients who were treated by a male physician had 1.8 times higher odds of receiving a psychoactive medication prescription. This means that an 82-year-old patient without dementia, depression, or restlessness has a 79% chance to be prescribed a psychoactive medication if she is female and the physician is male, compared with 61% chance for a female physician and a male patient without dementia. On the other hand, the age of residents lowered their odds of receiving a psychoactive medication by a factor of 0.967 per year (age). Dementia and depression increased the odds of receiving psychoactive medication by 1.74 and 5.15, respectively, while permanent restlessness increased the odds by 2.45.

A more detailed analysis showed that anxiolytic medications had higher odds of being prescribed by male physicians to female patients and antipsychotics had higher odds to be prescribed by younger physicians. Antipsychotics had 4.45 times higher odds to be prescribed to patients with permanent restlessness, compared with anxiolytics that had 2.54 times higher odds.

## Discussion

Our study, which included a representative sample of elderly nursing home residents, demonstrated that almost three fourths of nursing home residents in Slovenia had at least one prescription for psychotropic medication. The most problematic finding was prescribing of antipsychotics to patients with dementia; nearly one half of residents with dementia took antipsychotics. Also, prescribing of benzodiazepines in demented patients with agitation was frequent. On the other hand, most of the patients with depression were prescribed antidepressants. Prescribing of psychotropic medication depended mainly on the residents’ characteristics; residents taking psychotropic medication were also taking a greater number of all drugs.

Nursing homes are unique institutions, hosting the frailest segment of the elderly population, who are frequently exposed to inappropriate prescribing. The leading causes of psychotropic medication use in nursing home residents are dementia, depression, and sleeping disorders. The high prevalence of psychotropic medication use in our sample (73%) is comparable to results from other countries in Europe. The prevalence in Austria was 74.6% (21), in Finland 79.7% (20), in Switzerland 78.1% (23), and in Sweden 70.0% (24).

The most prevalent psychotropic medication group were hypnotics and sedatives, which were prescribed to almost half of the residents. The same results were reported by a study in Sweden (24), but other studies in that country found a lower prevalence of sedatives and hypnotics use, prescribed mainly for sleeping problems (20,21). Insomnia has been reported in 50% of chronically ill residents (24) and the aim of prescribing in insomnia is mainly improvement in the quality of residents’ life (31). In nursing homes, sleeping problems are more likely to be recognized and treated due to the residents’ (24), neighbors’, or staff’s requests. On the basis of the comparison between the literature data on the prevalence of insomnia in the population of chronically ill patients and our data on sedatives and hypnotic prescribing, we believe that in Slovenia short-acting sedatives and hypnotics have been mostly appropriately prescribed, with an aim to improve the residents’ quality of life.

Anxiolytics (excluding hypnotics and sedatives) were prescribed in a similar proportion of residents as in nursing homes in Austria – 22% (21) and in a slightly lower proportion than in Sweden – 31% (24) and Finland – 26% (20). In nursing homes in Slovenia, mostly low doses of short-acting benzodiazepines and less frequently long-acting benzodiazepines or high-doses of short-acting benzodiazepines were prescribed (28). Although the prescribing pattern of benzodiazepines in Slovenia is not among the most important reasons for potentially inappropriate prescribing according to Beers criteria (10), the proportion of nursing home residents taking benzodiazepines is too high, because benzodiazepines are frequently used for treatment of agitation in demented patients.

Depression was common – due to the old age, the presence of chronic diseases, and living in the institution (32). The percentage of prescribed antidepressants was similar to the percentage of residents taking antidepressants. In Slovenia, the first choice for depression treatment in primary care are SSRIs (sertraline, citalopram, s-citalopram, paroxetin). Prescription of tricyclic antidepressants, which can have anticholinergic and sedation properties in the elderly, or long acting SSRIs (fluoxetine), which could produce excessive central nervous stimulation, sleep disturbances, or increasing agitation in the elderly, was rare (fewer than 5% of all the patients who were taking antidepressant therapy) (28). Our findings suggest that if the diagnosis of depression was recognized, depression was also treated appropriately. However, previous studies have reported undiagnosed depression in nursing home residents (32,33), so it is possible that not all residents with depression were recognized and are not receiving appropriate treatment. Especially since residents with dementia manifesting psychical or verbal aggressive behavior were found to have a higher prevalence of depression than those not manifesting such behavior (32).

Antipsychotics were prescribed in 28% of elderly nursing home residents. The percentage is comparable to that in Finland (24) and the United States (29), but is lower than in Austria, where the rate of antipsychotic medications is extremely high (46%) (21).They explained the high rate with the inappropriate use of antipsychotics for treatment of sleeping disorders (21). Due to the high prescribing rate of hypnotics and sedatives in Slovenia and the findings that antipsychotics were prescribed more often in patients with dementia and permanently restless people, we assume that the main indication for prescribing antipsychotics in Slovenia is to control behavioral and psychological symptoms in patients with dementia, as confirmed in several other studies (18,34). High prescribing rate of antipsychotics may be the result of using the inappropriate indications, and presents the main source of prescribing errors in elderly nursing home residents. Even when antipsychotics have to be prescribed, it is essential to clearly document the indications for prescribing, reducing, and withdrawing such a drug.

The second aim of our study was to explore the residents’, physicians’, and nursing home characteristics associated with prescriptions of psychotropic medications. We found that the prescribing rate was mostly influenced by patients’ characteristics. Greater number of all prescribed drugs was an important patient’s characteristic that predicted prescribing of psychotropic medications. This is in accordance with other studies that also found that patients with a greater number of all prescribed drugs had more morbidities (35) and that the presence of physical illness was also a risk factor for psychical illness (36). Restlessness was the second important predictor of psychotropic medication prescribing. The same results were found in Austria (21), where antipsychotics were prescribed for patients with dementia-related wandering, pacing, and repetitive vocalization. This is also likely to be the case in Slovenia but in a smaller extent, due to the smaller proportion of prescribed antipsychotics. We found depression to be a predictor of anxiolytics prescription and dementia a predictor of antipsychotics prescription. The results of previous studies on depression and dementia as predictors are inconsistent (9,18,21).

Regarding nursing homes characteristics, all the included nursing homes are non-profit state-owned organizations and have the same standards for the staff/residents ratio, which was found elsewhere as a factor influencing antipsychotic prescription (37). Nursing homes with a smaller number of residents had a slightly lower prescribing rate of psychotropic medication, but the other nursing home characteristics did not influence the prescribing rate.

Physicians’ characteristics have been found to be associated with prescriptions and cost of drugs (38,39). In our study, male physicians prescribed psychotropic medications and anxiolytics more often. The same was found in a study in Canada (38), but an Italian study found greater prescription rate in female general practitioners (39). We also believe that regional disparities should be examined. It may be unfair to compare physicians without considering the location of their practice, but it is also improbable that all physicians who practice in a certain fashion aggregate in the same areas. We also found that younger physicians and physicians with less experience working in nursing homes prescribed more antipsychotics. In Slovenia, young and inexperienced residents in general practice are frequently sent to work in the nursing homes at the beginning of their careers (28), despite the fact that physician’s work in a nursing home is extremely demanding and involves dealing with many clinical and ethical dilemmas (40).

This is the first study in Slovenia that analyzed psychotropic medication prescribing in nursing homes. It included a large sample of randomly selected nursing home residents, which makes the results generalizable at the national level. However, it has some limitations. All of the physicians who participated in our study were members of the Elderly People Workgroup and therefore may be more motivated to work with elderly patients, discuss their problems, and prescribe fewer psychotropic medications. Also, we used the paper medical record as a source of data about functional status and diagnosis, which has some advantages (ethically acceptable, cheap, and easy to perform because of its retrospective design) but also the disadvantage of varying data quality depending on the physician (41). Also, some of the records lacked the data about functional status, which is why we were unable to classify all the residents into one of the three functional status categories. We did not collect the data about all the diagnoses with a potential indication for psychotropic drug prescribing. Although we used one of the best known tools for assessment of quality of prescribing in elderly patients – Beers criteria (10), we confirmed the previous findings that Beers criteria did not cover all the aspects of inappropriate prescribing (42,43). Therefore, it would make our conclusions on the quality of prescribing more grounded if we collected the data about other diagnoses that could be a potential indication for psychotropic medication prescribing. The data about the number of comorbidities could be also of our interest, since it is an important factor influencing the number of prescriptions (35) and psychotropic medication use.

There was no variation in the quality of data about medication prescribing, because nursing homes in Slovenia use the same medication prescribing software. We did not have enough data on the organizational structure of the nursing homes, which could be a factor of variation in psychotropic medication prescribing rate (37). Lack of such data could explain only a relatively small proportion of explained variability of psychotropic medication prescription in our logistic regression models. Another problem is that there are no national guidelines defining inappropriate prescribing of psychotropic medication, which would enable us to compare our findings with the perceived standards. For future research, it would be useful to define such standards and estimate the quality of prescribing according to them. Based on such comparison, the quality of prescribing could be reassessed and further interventions planned if the psychotropic medication prescribing was still a problem.
